# Posterior Circulation Stroke in a Patient With May-Thurner Syndrome and Patent Foramen Ovale: A Case of Paradoxical Embolism

**DOI:** 10.7759/cureus.95824

**Published:** 2025-10-31

**Authors:** Carlos A Suanes Zubizarreta, Michael Sinnott, Zehra Rizvi, Roberto Sanchez, Ariol Labrada

**Affiliations:** 1 Department of Translational Medicine, Florida International University, Herbert Wertheim College of Medicine, Miami, USA; 2 Department of Neurology, Palmetto General Hospital, Hialeah, USA

**Keywords:** antiphospholipid antibody syndrome (aps), may-thurner syndrome (mts), mr venography (mrv), paradoxical emboli, patent foramen ovale (pfo), posterior circulation stroke

## Abstract

Posterior circulation strokes can present with a range of subtle or fluctuating symptoms, often delaying diagnosis and complicating treatment decisions. We present the case of a 59-year-old woman with a prior history of transient ischemic attack (TIA), ischemic stroke, and antiphospholipid syndrome who was brought to the emergency department with acute dysarthria, vertigo, tinnitus, and vomiting. Imaging was initially unrevealing, but brain MRI confirmed acute infarcts in the superior vermis and right cerebellar hemisphere. Further workup revealed a patent foramen ovale (PFO) and May-Thurner syndrome (MTS), which were suspected contributors to a paradoxical embolic stroke. She underwent successful PFO closure and stent placement for left iliac vein compression. This case highlights the diagnostic challenge of posterior strokes and underscores the importance of considering underlying embolic and structural contributors in cryptogenic stroke presentations.

## Introduction

Cryptogenic stroke accounts for 30-40% of ischemic strokes in younger and middle-aged adults, yet the underlying mechanism often remains elusive [1]. Paradoxical embolism through patent foramen ovale (PFO) has emerged as an important and treatable cause in this population. PFO is present in about 25% of the general population and is more prevalent among patients with cryptogenic stroke [2]. The proposed mechanism of paradoxical embolism involves venous thrombi traveling through the right-to-left shunt, bypassing the pulmonary circulation, and embolizing systemically. High-risk features, including large shunt size or associated atrial septal aneurysm, further increase stroke risk. Randomized clinical trials, such as RESPECT, CLOSE, and REDUCE, have explored the success of PFO closure to reduce recurrent strokes in selected patients [3,4,5], and the 2021 American Heart Association (AHA) American Stroke Association (ASA) guideline recommends consideration of closure in carefully chosen cases [6].

When multiple embolic risk factors coexist, such as PFO, May-Thurner syndrome (MTS), and antiphospholipid syndrome (APS), diagnostic and therapeutic decision-making becomes particularly complex [2].

MTS, the extrinsic compression of the left common iliac vein by the right common iliac artery, predisposes to pelvic venous thrombosis and can provide a venous source for paradoxical embolism [7]. While MTS is a recognized risk factor for deep vein thrombosis, it is less commonly identified as a dual source of paradoxical embolic stroke. Routine lower extremity venous duplex often does not evaluate the iliac veins; advanced imaging modalities such as MR venography (MRV) or intravascular ultrasound (IVUS) are required for definitive diagnosis [8,9]. Case reports have described paradoxical embolic stroke in the setting of PFO and MTS co-occurrence [10,11].

Beyond structural cardiac and venous abnormalities, systemic hypercoagulable states further complicate stroke risk assessment. Antiphospholipid syndrome (APS) is an acquired autoimmune thrombophilia that further complicates stroke risk by creating a systemic prothrombotic state [12,13]. Current recommendations favor vitamin K antagonists over direct oral anticoagulants for secondary prevention in patients with APS and arterial thrombosis [12,13].

While PFO and MTS have each been individually linked to cryptogenic stroke, their co-occurrence with APS creates a unique clinical scenario where venous thrombosis risk, paradoxical embolism pathways, and systemic thrombophilia converge. We present a patient with a posterior circulation stroke and a unique convergence of APS, PFO, and MTS, underscoring the complexity of stroke mechanisms and the importance of considering all aspects of a patient's case [14].

## Case presentation

A 59-year-old woman with a medical history of antiphospholipid syndrome, dyslipidemia, transient ischemic attack (TIA) in 2009, and ischemic stroke in 2020 without residual deficits presented via EMS (emergency medicine services) to the emergency department (ED) following referral from an urgent care facility. At home, she had suddenly developed intense tinnitus in her left ear, followed by vertigo, nausea, vomiting, along with slurred speech, and difficulty finding words. EMS noted dysarthria, which had resolved by the time of ED arrival, but vomiting persisted. She denied recent trauma, bleeding, or surgeries.

**Figure 1:**
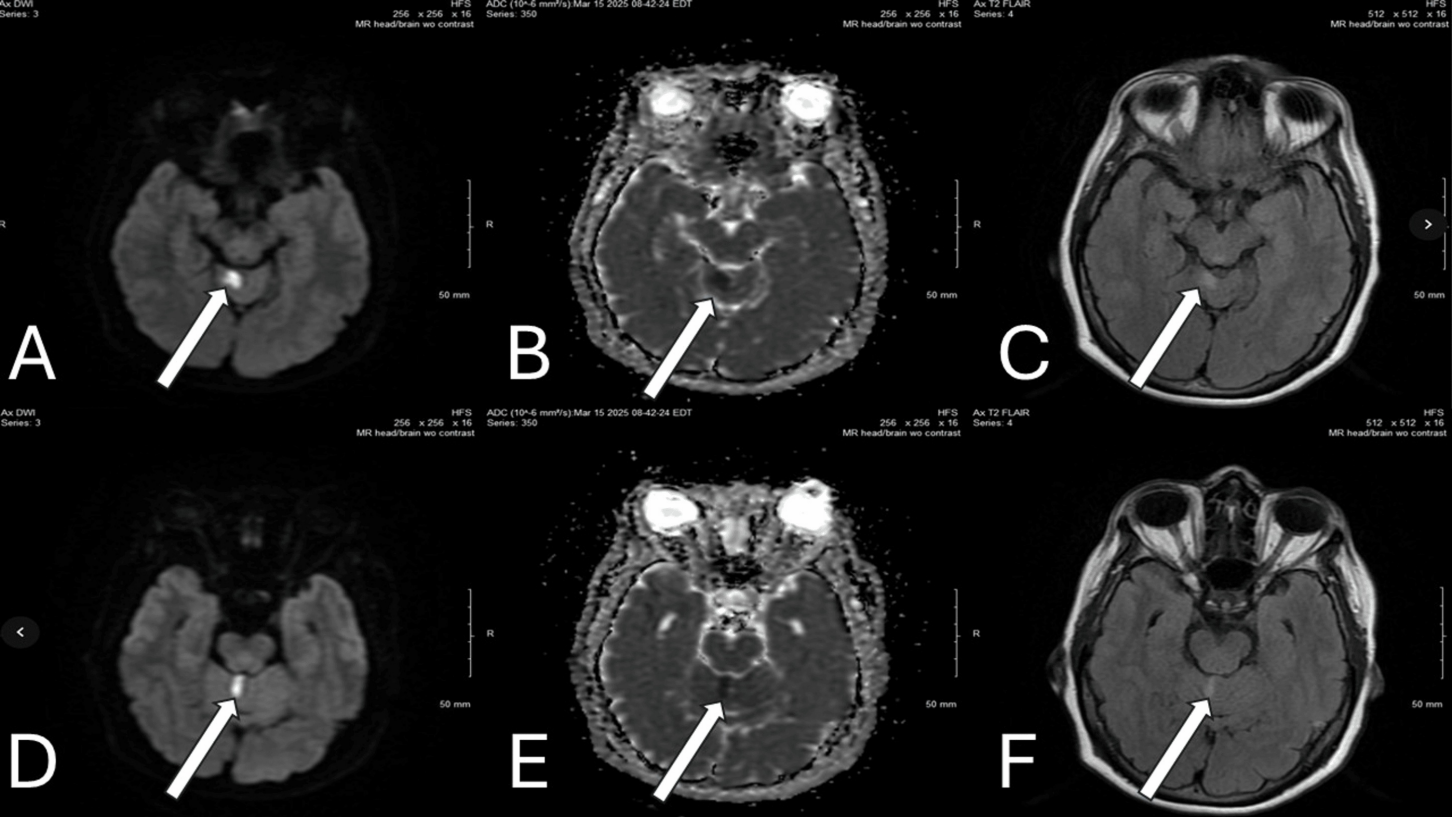
Brain MRI axial images (A and D: DWI, B and E: ADC, C and F: T2 FLAIR) showing small foci of diffusion restriction in the right superior vermis. Hyperintense signals are appreciated on the T2 FLAIR and DWI with hypointense signals on ADC, indicative of an acute infarct. Arrows are pointing to the acute infarcts. ADC: apparent diffusion coefficient, DWI: diffusion-weighted imaging, MRI: magnetic resonance imaging, T2 FLAIR: T2-weighted fluid-attenuated inversion recovery

On presentation, her vital signs were stable: blood pressure was 124/97 mm Hg and glucose was 114 mg/dL. A stroke alert was activated with a National Institutes of Health Stroke Scale (NIHSS) score of zero at the time of evaluation in the ED. Despite the benign NIHSS score and negative initial CT imaging, the persistent and acute onset of severe vestibular symptoms (vertigo, tinnitus, nausea, and vomiting) maintained a high clinical suspicion for posterior circulation stroke, justifying the need for advanced imaging. Head CT and CT angiography showed no acute intracranial abnormality or large vessel occlusion. CT perfusion demonstrated no mismatch or penumbra. MRI of the brain revealed small, acute infarcts in the superior vermis and right cerebellar hemisphere (Figure 1). Table 1 shows the summary of key findings and interventions.

**Table 1:** Summary of key findings and interventions APS: antiphospholipid syndrome, ASA: atrial septal aneurysm, GPi: glycoprotein I (referring to β2​-glycoprotein I), IVUS: intravascular ultrasound, LVEF: left ventricular ejection fraction, MRI: magnetic resonance imaging, MRV: magnetic resonance venography, MTS: May-Thurner syndrome, NIHSS: National Institutes of Health Stroke Scale, PFO: patent foramen ovale, RoPE: risk of paradoxical embolism, TIA: transient ischemic attack

Category	Finding/measurement
Patient profile and presentation	
Age	59-year-old woman
History	Antiphospholipid syndrome (APS), TIA (2009), ischemic stroke (2020)
Acute symptoms	Persistent vertigo, intense tinnitus, nausea, vomiting, transient dysarthria
NIHSS score	Zero (on ED evaluation)
Stroke location (MRI)	Small, acute infarcts in the superior vermis and right cerebellar hemisphere (Posterior Circulation)
Cardioembolic source (PFO)	
PFO description	Large PFO
Associated high-risk features	No atrial septal aneurysm (ASA) found
LVEF	Normal
RoPE score	5 (34% probability stroke is PFO-related)
Venous source (MTS)	
MRV finding	Approximately 50% compression of the left common iliac vein
IVUS confirmation	84% stenosis of the proximal left common iliac veins
MTS intervention	Placement of a 14 x 100 mm bare steel self-expanding stent
MTS outcome	Resolution of collateral emptying and no residual stenosis
Systemic hypercoagulability (APS)	
Diagnosis	Maintained based on prior positive testing
Acute lab results	β2​-glycoprotein I (GPi) IgG and IgM: within normal limits; anticardiolipin IgA: <9
Long-term management	
Discharge anticoagulation	Warfarin (consistent with guideline-directed care for APS with arterial thrombosis)

**Figure 2:**
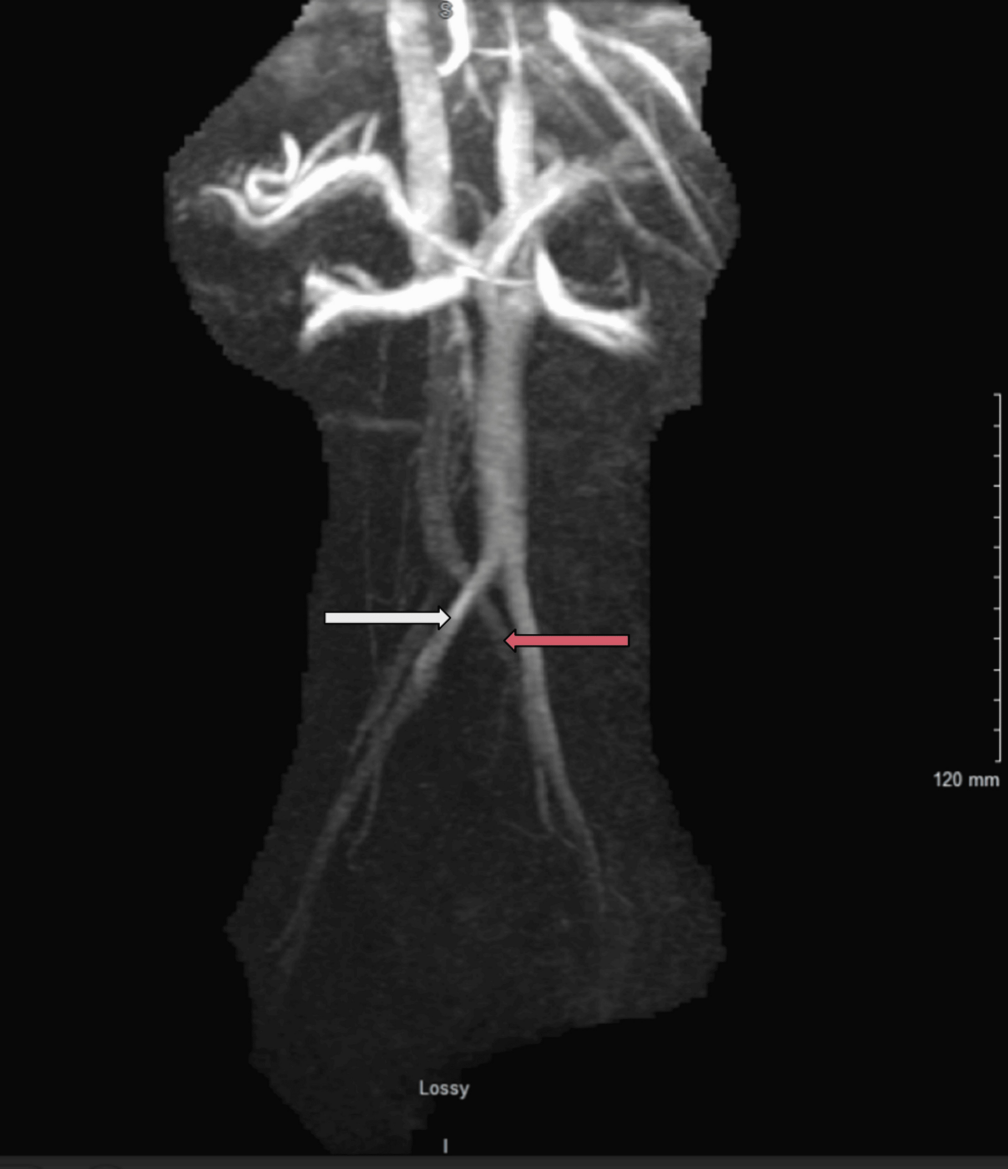
MRV pelvis indicating 50% compression of the left common iliac vein (red arrow) between the right common iliac artery (white arrow) and lumbar vertebra, immediately proximal to the IVC confluence compatible with May-Thurner syndrome. IVC: inferior vena cava, MRV: magnetic resonance venography

A cardioembolic workup, which included echocardiography, revealed a large PFO. The study confirmed a normal left ventricular ejection fraction (LVEF) and no evidence of an atrial septal aneurysm. The patient underwent successful closure of the defect by the interventional cardiology team during this admission. Addressing the patient's history of APS, the hypercoagulable workup performed during this admission revealed beta 2-glycoprotein I IgG and IgM and anticardiolipin antibodies within normal limits. However, the clinical diagnosis of APS, based on prior positive APA testing, was maintained. In addition, MR venography (MRV) of the pelvis revealed approximately 50% compression of the left common iliac vein between the right common iliac artery and lumbar vertebra, consistent with MTS (Figure 2). Intravascular ultrasound during interventional radiology evaluation confirmed 84% stenosis of the proximal left common iliac vein. A 14 x 100 mm bare steel self-expanding stent was placed in the left common iliac vein during this admission. Post-procedure angiography confirmed resolution of collateral emptying and no residual stenosis. 

During hospitalization, the patient’s neurological deficits largely resolved. She was fully alert, oriented, interactive, and able to follow commands. She was discharged on warfarin with optimized medical therapy and scheduled follow-up.

## Discussion

MTS arises when the right common iliac artery compresses the left common iliac vein, producing anatomic narrowing and endothelial injury [7,14]. Chronic endothelial injury promotes intimal hyperplasia and intraluminal fibrosis, which further narrows the lumen and alters venous hemodynamics, as described in classic surgical series [15]. The resulting venous stasis and endothelial injury fulfill Virchow’s triad and predispose to left-sided iliofemoral thrombosis. The risk is further amplified by additional prothrombotic factors such as hormonal exposure or thrombophilia [7,16]. Routine lower extremity duplex ultrasound misses proximal iliac vein disease; MRV or IVUS is required for definitive diagnosis.

In the presence of a PFO, thrombi generated in the venous system may travel across the interatrial shunt and embolize to the cerebral circulation. This mechanism provides a plausible paradoxical embolic mechanism for otherwise cryptogenic stroke; however, it is difficult to name this as the exact cause with complete certainty, as there could be other sources of embolism [10,11].

In addition to venous outflow obstruction and paradoxical embolism, this patient had antiphospholipid syndrome (APS) and acquired autoimmune thrombophilia that increased the risk of both venous and arterial thrombosis. APS is strongly associated with recurrent ischemic stroke and other systemic arterial events. The European League Against Rheumatism (EULAR) recommendations advise long-term anticoagulation with vitamin K antagonists rather than direct oral anticoagulants (DOACs) due to higher recurrence risk [12,13]. This further complicates management of our case, as PFO closure and iliac vein stenting alone would not address the systemic hypercoagulable state of APS in this patient. The decision to continue warfarin after structural interventions was consistent with guideline-directed care. 

Case reports of cryptogenic stroke associated with PFO and MTS are rare. Phelps et al. (2019) described a patient with cryptogenic stroke, with MTS detected by MRV and PFO closure plus iliac stenting leading to a favorable outcome [10]. Zoltowska et al. (2018) similarly reported paradoxical embolism in a patient with PFO and MTS [11]. Our case is unique in that APS was also present, fully fulfilling Virchow’s triad and altering typical management. This combination increases the risk of recurrence and underscores the importance of a broad multidisciplinary work-up and management. 

Most paradoxical embolic strokes due to PFO are described in the anterior circulation, often affecting the middle cerebral artery distribution. This is supported by computational studies showing that larger emboli preferentially travel into the MCA due to vessel caliber, geometry, and flow partitioning [17,18]. However, posterior circulation involvement is documented in The New England Medical Center Posterior Circulation Stroke Registry, which states that embolism is a common mechanism of vertebrobasilar territories [19]. This pattern of embolism is more common with larger right-to-left shunts [20,21]. A multicenter cohort analysis found that posterior circulation alone was not significantly different in cardioembolic versus non-cardioembolic strokes, emphasizing that vascular territory alone should not be used to determine mechanism [22]. Our patient developed small acute infarcts in the superior vermis and right cerebellum. This case highlights that paradoxical embolism should be considered even in posterior circulation presentations. 

The Risk of Paradoxical Embolism (RoPE) score is a validated tool estimating the probability that a stroke is PFO-related. It looks at the patient's history of hypertension, diabetes, stroke or TIA, smoking, age, and presence of a cortical infarct on imaging to calculate risk [23]. Our patient’s RoPE score was 5, indicating a 34% chance that the stroke is due to PFO and warrants further workup. Importantly, RoPE does not incorporate concurrent prothrombotic states such as APS or MTS. This emphasizes that RoPE scores can be used to guide decision-making, but should not be viewed as an absolute determination of care. Pelvic venous imaging is not routinely part of cryptogenic stroke evaluation, as demonstrated by Osgood et al. However, 18% of patients with cryptogenic stroke and PFO had abnormal pelvic MRVs, including 10% with MTS diagnosed [24]. Selective use of MRV or IVUS in cryptogenic stroke may uncover an underrecognized venous source of embolism. Multidisciplinary collaboration between neurology, cardiology, hematology, and interventional radiology was essential in this case, and larger prospective studies are needed to determine recurrence risk and guide management for patients with overlapping embolic risk factors. It must be acknowledged that this report is limited by its single case nature, restricting the generalizability of the findings regarding recurrence risk and long-term outcomes for patients with this specific overlap of APS, PFO, and MTS. Prospective studies are needed to address these uncertainties. 

## Conclusions

This case illustrates how concurrent APS, PFO, and MTS can converge to produce paradoxical embolism resulting in posterior circulation stroke. Multimodal imaging identified both intracardiac and pelvic venous sources of paradoxical embolism. She underwent successful closure and stenting, with good recovery. This case highlights the rare convergence of APS, PFO, and MTS, underscoring the multifactorial etiology of cryptogenic stroke and emphasizing the importance of comprehensive vascular evaluation, including pelvic venous imaging, to guide individualized multidisciplinary care. 
